# Neutrophil extracellular traps primed intercellular communication in cancer progression as a promising therapeutic target

**DOI:** 10.1186/s40364-023-00463-y

**Published:** 2023-03-02

**Authors:** Bingqing Shang, Honglei Cui, Ruiyang Xie, Jie Wu, Hongzhe Shi, Xingang Bi, Lin Feng, Jianzhong Shou

**Affiliations:** 1 Department of Urology, National Cancer Center/National Clinical Research Center for Cancer/Cancer Hospital, Chinese Academy of Medical Sciences and Peking Union Medical College, Panjiayuan Nanli 17# Chaoyang District, Beijing, 100021 PR China; 2 Department of Etiology and Carcinogenesis, State Key Laboratory of Molecular Oncology, National Cancer Center/National Clinical Research Center for Cancer/Cancer Hospital, Chinese Academy of Medical Sciences and Peking Union Medical College, Beijing, PR China

**Keywords:** Cancer, Neutrophil extracellular traps, Intercellular communication, Immune landscape, Biomarker, Therapeutic target

## Abstract

In addition to the anti-infection response, neutrophils are linked to tumor progression through the secretion of inflammation components and neutrophil extracellular traps (NETs) formation. NET is a web-like structure constituted by a chromatin scaffold coated with specific nuclear and cytoplasmic proteins, such as histone and granule peptides. Increasing evidence has demonstrated that NETs are favorable factors to promote tumor growth, invasion, migration, and immunosuppression. However, the cell–cell interaction between NETs and other cells (tumor cells and immune cells) is complicated and poorly studied. This work is the first review to focus on the intercellular communication mediated by NETs in cancer. We summarized the complex cell–cell interaction between NETs and other cells in the tumor microenvironment. We also address the significance of NETs as both prognostic/predictive biomarkers and molecular targets for cancer therapy. Moreover, we presented a comprehensive landscape of cancer immunity, improving the therapeutic efficacy for advanced cancer in the future.

## Introduction

Neutrophils are the most abundant type of leukocytes in human peripheral blood serving as the front line in innate immunity [1]. Besides infections, ongoing efforts have expanded the roles of neutrophils in a wide range of human diseases, such as thrombosis, auto-immune diseases, pulmonary diseases, and cancer progression [2]. The contribution of tumor-associated neutrophils (TANs) in cancer remains elusive, as a result of the complex microenvironment of cancer [3]. Research using animal models has shown that TANs can be polarized into an anti-tumor (N1) or pro-tumor (N2) subtype, which is driven by the state of TGF-β [4].

In 2004, Brinkmann et al*.* discovered that neutrophil suicide could release web-like structures decorated with depolymerized chromatin and antimicrobial molecules, named as NETs. They elicited NETs using PMA and IL-8 in vitro and demonstrated that NETs could resist virulence factors and kill bacteria [5]. Apart from PMA and IL-8, additional stimuli were found to be responsible for the release of NETs, including protozoa, bacteria, fungi, IFN-α/IFN-γ/C5a, GM-CSF/C5a, lipopolysaccharide (LPS), antibody-antigen complexes, activated platelets, and calcium ionophores [6]. Within NETs, there are several proteins coated in the decondensed DNA, such as histones, neutrophil elastase (NE), myeloperoxidase (MPO) and cathepsin G [7–9].

Like the role of TANs, increasing evidence has recently revealed the role of NETs as a fundamental part in cancer progression [10–12]. The prior reviews mainly focused on the crucial role of NETs in contributing to the development and progression of cancer. Consistent with the previous studies, our research validated the role of NET as an unfavorable prognosis biomarker in most types of cancer, by developing a panel of genes signature as NET-score [13]. Nevertheless, this review first summarizes the intercellular communication between NETs, tumor cells, and immune cells in carcinogenesis. We aimed to figure out the cellular interplay and molecular pathway mechanism that regulate the innate immune and adaptive immune response in malignancies. The potential of NETs as diagnostic and prognostic markers and novel treatment targets is also discussed in this review.

### The interplay between the tumor cell and NETs

The role of NETs in pro- and anti-tumorigenic functions remains unclear in different cancers. Very few articles now illustrated the in-vitro antitumor effect of NETs. An experiment in vitro showed that NETs could impede growth and induce apoptosis in colorectal cancer cells [14]. Consistently, Schedel F et al*.* found NETs inhibiting the migration and necrosis of melanoma cells in vitro [15]. However, NETs in the tumor microenvironment (TME) mostly exert promotion in the growth and progression of cancer, in the onset and spread of tumor metastasis, and in the poor response of anti-tumor therapy [12, 16–23]. The bond between cancer-cells and NETs was revealed in the Ewing sarcoma at first, in which the NETs were correlated with poor prognosis for patients [12]. Compared to control mice, the neutrophils in the tumor-bearing mice represented an increased ability to spontaneously form NETs [24, 25], which was associated with endothelial-to-mesenchymal transition (EMT) driving and cancer metastasis [25–27]. The cancer cell-derived factors supporting the NETosis include mainly IL-8, G-CSF, GROα/β, and CXCR1/2 chemokine receptor agonists [17, 28]. NETs also strengthen the metastatic potential of cancer cells by other mechanisms, including accumulation in the pre-metastatic niche or the circulation for entrapping the circulating tumor cells (CTCs) [17, 29].

Szczerba BM et al*.* manipulated single-cell RNA sequencing (scRNA-seq) and investigated the ligand/receptor pairs of the CTC–neutrophil cluster. They hypothesized that the expression of VCAM1 is a molecular feature possibly defining the CTC-neutrophil cluster formation in breast cancer [30]. Understanding the ligand/receptor pairing may explore new targets in cancer therapy.

Recently, several pieces of research demonstrated the molecular mechanism of the intracellular network of tumor cells and NETs. The most studied example of the ligand/receptor interaction was the TLR family in the tumor cell and NET-associated factors. The TLR family is characterized as the essential part of the innate immune and could recognize pathogen-associated molecular patterns (PAMPs) [31]. TLR receptors are ubiquitously expressed both in tumor and immune cells [32, 33], and exert a dual role in cancer [34–37]. To date, several TLR agonists have shown inspiring results for their survival benefits combined with immune vaccination, immune checkpoint inhibitors, and chemotherapy in clinical trials, especially for glioma [38, 39]. Nonetheless, TLR agonist-associated infection and the help of TLR overexpression in the carcinogenesis or tumor progression should be emphasized [33, 36, 40]. Higher levels of intratumor NETs and preoperative serum MPO-DNA as a marker of NETs were correlated with shorter survival in metastatic colorectal cancer. Mechanistically, NE as NETs-derived stimulatory factor directly activated the TLR4 pathway on tumor cells and subsequently upregulated Peroxisomes proliferator-activated receptor gamma coactivator 1-alpha (PGC1-α), driving mitochondrial homeostasis and favoring the tumor growth [41]. In bladder cancer, Shinde-Jadhav S et al*.* found the level of NETs was increased after radiation therapy (RT), which contributed to tumor radiotherapy resistance. They further demonstrated that the activated formation of NETs was associated with HMGB1 via a TLR4-dependent manner, and inhibiting NETs or HMGB1 could improve radiation response [23]. For diffuse large B-cell lymphoma (DLBCL) patients, Nie M et al. investigated the mechanism of interleukin-8 (IL-8), secreted by lymphoma cells, binding to C-X-C Motif Chemokine Receptor 2 (CXCR2) on the cell-surface of neutrophil and inducing NET formation. Furthermore, an increased level of NETs activated TLR9 on the lymphoma cells, contributing to NFkB, STAT3, and p38 downstream pathways activation. The novel cross-talk as IL8-CXCR2-TLR9 axis augmented the tumor progression in DLBCL [21]. Tohme et al*.* proposed that HMGB1 released from NETs assisted in the TLR9 activation. TLR9 promoted colorectal cancer cell proliferation, migration, or invasion by activating the MAP kinase pathways [42]. The neutrophils derived from metastatic hepatocellular carcinoma (HCC) harbored an up-regulated capacity of producing NETs, compared with those in healthy adults. It was further investigated that NETs enhanced the invasion capacity of trapped tumor cells through the activation of the TLR4/9 receptor and the phosphorylation of P65 and cyclooxygenase-2 (COX2) overexpression. The direct inhibition of the TLR4/9-COX2 pathway wrecked the NET-driven metastatic potential [43]. Apart from the TLR ligand-associated pathway, CCDC25 is a transmembrane protein on the breast cancer cells, which could interact with the NET-DNA complex directly. As a result, it enhanced tumor cell motility and tumor metastasis by activating the downstream pathway including integrin-linked kinase (ILK) and β-Parvin. CCDC25-knockout cells abrogated the NET-mediated potential metastasis [11]. Cell-to-cell adhesion in cancer is complex and involved in each step of tumor progression. It enables tumor cells to loosen from the primary tumor mass and enhances cell attachment to the metastatic site [44, 45]. In the cell adhesion process, the integrin ligands (a combination of α and β subunits) determine the central role governing cancer cell migration [44]. In a panel of tumor cell lines, Monti M et al*.* revealed that NETs could adhere to tumor cells with high levels of integrin α5β1, αvβ3, and αvβ5 [46]. Tumor-derived integrin β1 promoted the co-localization of NETs and tumor cells in vivo and in vitro. Najmeh S et al*.* proposed the hypothesis that NETs captured CTC through the mediator integrin β1 [29]. Mechanism research demonstrated that NETs-related proteases, matrix metalloproteinase 9 (MMP9) and NE, resulted in the cleavage of laminin. The NET-remodeled laminin-111 subsequently activated the integrin a3β1 receptor on the tumor cell. The integrin a3β1 up-regulated the focal adhesion Kinase (FAK), extracellular signal-regulated kinase (ERK), and yes-associated Protein (YAP) and promoted the dormant tumor cell awaken [10].

In conclusion, there is growing evidence from ligand/receptor pair analysis that NETs primarily promote the proliferation, adhesion, and metastatic capacity of tumor cells (shown in Table 1). Meanwhile, the molecular mechanism of its anti-tumorigenic effects requires further exploration. NETs directly or the NETs-associated factors interact with the receptors on the tumor cells and thus alter the tumor cell function. Nevertheless, there are few studies on the mechanism of interplay between tumor cells and NETs, especially for their ligand-receptor pairs. Furthermore, targeting the ligand-receptor pairing or specific kinases rather than neutrophils or tumor cells could be a potential strategy for anti-tumor treatment.

**Table 1:** The interplay between the tumor cell and NETs

Stimulator or Ligand of NETs	Receptors in the tumor cell	Regulated function	Type of Cancer	Reference
IL-8, G-CSF, GROα/β	-	tumor premetastatic niche formation	ovarian cancer	[17]
CXCR1/CXCR2 Agonists	-	immune-mediated cytotoxicity	solid malignancies	[28]
NE	TLR4	mitochondrial biogenesis and tumor growth	Colorectal cancer	[41]
HMGB1	TLR4	radioresistance	bladder cancer	[23]
NETs	TLR9	tumor proliferation, migration, and invasion	Colorectal cancer	[42]
NETs	TLR9	tumor proliferation and metastasis	Diffuse large B-cell lymphoma	[21]
NETs	TLR4/9	tumor metastasis	Hepatic cell carcinoma	[43]
NET-DNA complex	CCDC25	tumor cell motility and tumor metastasis	Breast cancer	[11]
NETs	integrin α5β1, αvβ3, and αvβ5	tumor cell adhesion	Pan-cancer	[46]
NETs	integrin β1	tumor cell adhesion	Lung cell carcinoma	[29]
NETs-cleaved laminin-111	integrin a3β1	awaken dormant tumor cell	Breast cancer	[10]

### Potential interactions between the macrophage and NETs

Macrophages polarize to activated pro-inflammatory M1 and anti-inflammatory M2 phenotypes depending on the microenvironment stimuli [47, 48]. M1 macrophages exert pro-inflammatory effects through secreted cytokines, such as interleukin-1β (IL-1β), interleukin-6 (IL-6), and tumor necrosis factor (TNF). In contrast to M1 phenotype macrophages, M2 macrophages are predominantly correlated with resolving inflammation and promoting tissue repair [49]. Tumor-associated macrophages (TAMs) are typically altered into M2 and mediate immune dysfunction in the TME [50]. Similar to neutrophils, macrophages also release web-like structures as extracellular traps (ETs) [51]. Macrophage extracellular traps (METs) exert tumor-promoting roles by assisting tumor growth, progression, and metastasis [51–53]. For pancreatic neuroendocrine tumors (pNETs), NETs and METs were deemed as the independent prognosis indicators for recurrence-free survival (RFS). However, no significant correlations were founded between NETs and METs in this research. It was postulated that NETs and METs were regulated by different mechanisms [54]. Zhang L et al*.* delineated the ability of NET to induce the migration and invasion of lung adenocarcinoma cells in vitro, which is partly dependent on macrophages [55]. Up to now, the molecular mechanism between NETs, macrophages, and METs is not fully described in cancer. To spur new ideas in cancer, we briefly introduced the recent studies, which uncovered the NETs-macrophage interaction in non-neoplastic diseases [56–58].

In response to infection, the caspase-1 dependent cell death pyroptosis is another regulated defending way in addition to generating ETs [59]. The novel mechanism was identified as NET-related HMGB1 activated the receptor for advanced glycation end products (RAGE) pathway signaling and subsequently trigger macrophage pyroptosis in sepsis [56]. Li H et al*.* indicated the interaction between NETs and macrophage pyroptosis, aggravating the inflammation of acute respiratory distress syndrome (ARDS) [60]. The inflammatory microenvironment plays an important role in all stages of tumor development and progression. It is necessary to further understand whether the interaction between NET and macrophages has the effect of amplifying the inflammatory response within the tumor and accelerating tumor progression.

A series of research investigated whether NETs could induce a cellular response in macrophage differentiation. After stimulation with low-density granulocyte (LDG)-derived NETs in the coronavirus disease 2019 (COVID-19), macrophages were characterized by supernatant proinflammatory cytokines secretion [61]. The role of NETs-related macrophage inflammatory phenotype polarization was similar in diabetic mice, contributing to atherosclerosis progression. Apart from the inflammasome-associated markers overexpression in NETs^+^ regions, the upregulation of the glycolytic pathway also symbolized a shift toward an M1-like phenotype in the atherosclerosis plaque area [62, 63]. EGF-like repeats and discoidin I-like domain 3 (EDIL3) were previously reported in the inflammatory regulation and neutrophil recruitment inhibition, through the interaction with lymphocyte function-associated antigen 1 (LFA1) or intercellular adhesion molecules (ICAMs) [64, 65]. EDIL3 was negatively associated with neutrophil recruitment and macrophage expansion in myocardial infarction (MI). The DNA moiety of NETs then licensed a switch towards M1-like macrophage polarization in the deficiency of EDIL3. Researchers further validated the mechanistic evidence that NETs induced inflammatory macrophage polarization via the TLR9 pathways, exerting DNA sensors to transduce NETs-macrophage interactive signals [66]. The evidence described above shows that the ubiquitously expressed TLR receptors are an important bridge for mediating NET and tumor cell interaction for cancer Therefore, it is crucial to understand whether TLR receptors are involved in mediating NET and macrophage interactions in tumor diseases. After inflammation declined in the late stage of wound healing, NETs-treated anti-inflammatory macrophages initiated the fibrotic cascades, resulting in postoperative epidural fibrosis formation [67]. Taken together, NETs may exert a dual role in the function switch of macrophages.

Based on the finding that the macrophage amount was negatively correlated with the localized NETs density for patients with abdominal aortic aneurysm, Haider P et al*.* hypothesized that macrophage may clear the NETs in vivo [68]. NET clearance refers to the phagocytosis of the NET by macrophage. Apart from the inflammatory macrophage phenotype polarization, aggregation of NETs with reduced clearance by macrophages may give rise to an ongoing inflammatory response. Mechanistically, MMP12 was validated as a key mediator for macrophages to remove NETs, preparing for inflammation degradation and restoring immune homeostasis [69]. Furthermore, AMPK-associated pathway activation is also an important mechanism for NET clearance. Chiang et al*.* explored the mechanism that the 13-series resolvins (RvTs) enhanced NETs clearance by macrophages through cyclic adenosine monophosphate (cAMP) / protein kinase A (PKA) / AMP-activated protein kinase (AMPK) axis, providing a molecular mechanism for inflammation resolution [58]. It was further illustrated that the restoration of AMPK in macrophages could recover the NET clearance ability [70].Additionally, the inhibitors preventing NETs formation abrogated the proinflammatory macrophage recruitment, macrophage pyroptosis, M1-like phenotype polarization, and macrophage-associated NET clearance [60, 62, 70–73].

This evidence identified that the crosstalk between NETs and macrophages was involved as a fundamental part of non-neoplastic disease progression. There is a lack of cell–cell interaction analysis between NET and macrophages in cancer. However, the above results of these studies in non-neoplastic disease provided potent interest or research ideas for the tumor in the future. Further questions remain as: Does NET-associated inflammation influences macrophage function in cancer? What is the mechanism for the cellular mechanism for NETs-macrophage interaction in cancers? Could NETs-macrophages molecular network be a potential novel treatment target for the tumor?

### Cross-talk between NETs and lymphocytes

Lymphocytes including T lymphocytes, B lymphocytes, and natural killer (NK) cells, serve as a crucial mechanism to mediate the immune system hemostasis and regulate immune tolerance [74–76].

Recent findings in cancer demonstrated that tumor-specific lymphocytes primarily presented a dysfunctional state, shaped by the immunosuppressive tumor microenvironment, and thus promoted tumor escape and therapy resistance [75, 77, 78]. Especially T lymphocytes and NK cells exerted a fundamental part in tumor development and progression. There is increasing evidence that the complex interaction between NETs and lymphocytes may critically involve the immune function regulation in the tumor.

First, NETs exerted an inhibitory role in the amount and function of CD8^+^ T cells. The infiltrating rate of CD8^+^ T lymphocytes was inversely associated with the NETs density in human solid tumors including non-small cell lung cancer (NSCLC) and bladder cancer (BC) [79]. Teijeira Á et al*.* illustrated that the motility of CD8^+^ T cells migrating across the transwell was directly weakened by NETs in vitro[28]. Apart from the cell migration motility, Kaltenmeier C et al*.* investigated whether NETs could mediate the T cell dysfunction and exhaustion responses. In the NETs-rich TME, the tumor-infiltrating CD8^+^ T lymphocytes were characterized with functional exhausted phenotype, expressing high levels of exhaustion markers, such as PD-1, LAG-3, or TIM3. The direct modulatory role of NETs on CD8^+^ T cell's exhaustive differentiation was validated in the co-culture experiment. By co-culturing the NETs and CD8^+^ T cells in vivo, the exhausted phenotype changes of CD8^+^ T cells were as same as that in the NETs-rich TME. This phenotype shift was further reversed with the NETs inhibitor [80]. Second, NETs were illustrated for their positive correlation with CD4^+^ T cell exhausted phenotype differentiation and Foxp3^+^ regulatory T cells (Tregs) density [80, 81]. After the co-culture with NETs, the changes in naïve CD4^+^ T cells that could differentiate into Tregs, which presented with activated mitochondrial oxidative phosphorylation (OXPHOS) pathway. Naïve CD4^+^ T cells up-regulated the Treg-associated markers, including TGFB1, ID3, and DUSP4. Meanwhile, effector T cell-related genes in the program of effector T cell (Teff) differentiation were reduced [81]. It has been recognized that naïve CD4^+^ T cell differentiation depends on the balance of glycolysis and oxidative phosphorylation (OXPHOS) [82]. T cells in the NETs-rich area were presented with the down-regulated functioning mitochondria, reduced glucose but up-regulated fatty acid uptake [80]. The OXPHOS inhibitor reversed NETs-associated Treg differentiation [81]. Consistent with the previous study [83], the TLR4 on the naïve CD4^+^ T cell exerted an essential role in mediating the Treg activation and function. It was delineated that NETs directly contacted naïve CD4^+^ T cells mostly through TLR4, thus prompting Treg differentiation [81].

Nevertheless, these studies mentioned above merely considered the interrelationship between T cells and NETs in cancer. The relationship between NETs and B cells is not well understood. Most of the studies in this area are concerned about autoimmune diseases. Recent research reported that B cells were drivers of chronic inflammation in Rheumatoid arthritis (RA). Activated B cells were capable to release IL-8 recruiting neutrophils to the synovium, and produce autoantibodies activating the complement pathway and promoting NETs formation [84]. Additionally, citrullinated histones in NETs acted as a continuous source of fresh antigens to B cells [85]. In Systemic Lupus Erythematosus (SLE), uptake of NETs in Lupus Nephritis (LN-NETs) by B cells was found, and LN-NETs could also stimulate Naïve B Cells to produce IgG2 in SLE [86]. Correlating with disease severity, spontaneous NETs formation was enhanced by circulating neutrophils in Bullous pemphigoid (BP) patients [87]. Mechanism research showed that NETs formation could be abrogated by blocking Fcγ receptor and/ or NADPH pathway. Additionally, elevated levels of NETs in BP patients triggered B cells differentiation into plasma cells, producing a large amount of autoantibody, and this procedure was mediated by the activation of MAPK P38 cascade [87].

The situation becomes more complicated when considering the relationship between NET, lymphocytes, tumor cells, and others. It was postulated that the anti-tumor effect of lymphocytes was compromised by the reduced contact with NETs-shielding tumor cells [28], yet, the relevant molecular interaction mechanisms need to be further explored. In conclusion, the existing studies mostly focused on T lymphocytes, and NETs had a regulatory mechanism on the T lymphocytes infiltrating and functioning in cancer. The effects of NETs on NK cells and B cells are poorly understood and deserve further investigation.

### A positive feedback cycle between NETs and platelets

In addition to their well-known role in coagulation and hemostasis, accumulating evidence shows that platelets also exert a regulative role in the immune system [88–90]. The level of NETs is elevated in different cancers, including colorectal cancer (CRC), gastric cancer (GC), oral squamous cell carcinoma** (**OSCC), as well as pancreatic tumors, and the inhibition of NETs diminishes the hypercoagulability in cancer [91–94].

Increasing evidence has illustrated the role of platelet in NETs formation. Clark et al*.* first described platelet involved in DNA extracellular trap formation in a mouse model of sepsis. They found that platelets, activated by LPS through TLR4, could bind neutrophils and lead to their activation and NETs formation [95]. One potent mechanism of platelets-induced NETs formation seems to be the combination of P-selectin to its receptor P-selectin glycoprotein ligand-1 (PSGL-1) on their surface [96]. Animal studies demonstrated that platelets from mice with overexpressed P-selectin were more prone to generate NETs when co-incubated with neutrophils, while platelets from P-selectin knock-out mice failed to induce NETs [97]. Moreover, using anti‐P‐selectin and PSGL‐1 antibodies to abrogate the interaction between neutrophils and platelets could remarkably decrease NETs formation in the plasma of glioma patients [98]. The pro-inflammatory molecule platelet-derived high mobility group box 1 (HMGB1), secreted from activated platelets has also been shown to facilitate NET formation [99]. According to this study, platelets from colorectal cancer patients stimulated neutrophils to release NETs, which could be abolished by the absence of HMGB1 [92]. Meanwhile, platelets acted as carriers of tumor-derived exosomes, which in turn contributed to the generation of NETs [90].

It was shown that platelets promote neutrophils to generate NET and its components,which in turn activate platelets as wel l[100]. NETs could function in procoagulant response by providing a scaffold for platelets, red blood cells, extracellular vesicles, and pro-coagulant molecules [101–103]. NETs could convert platelets to a procoagulant phenotype and stimulate the activation and aggregation of platelets by upregulating phosphatidylserine and P-selectin expression on its membrane [98, 104]. Brian A. Boone et al*.* found that DNA and its receptor for advanced glycation end products (RAGE) were necessary for NETs-relevant platelet aggregation and RAGE KO tumor-bearing mice exhibited decreased platelet aggregation [93]. Another study showed that DNase I treatment could attenuate platelet aggregation, while some platelets still adhered to the glass slides. Histones that are the most abundant proteins in NETs or NE are sufficient to induce platelet aggregation [104]. Co-culture platelets with histones H3 and H4 promote its aggregation, whereas histones 1H, H2A, and H2B had no such effect [102]. More specifically, histone-enhanced platelet aggregation by recruiting fibrinogen and histone-dependent platelet activation seems to be mediated by the signaling pathway of TLR2 and TLR4 receptors, via the transcription factor NF-κB [105].

Holistically, activated platelets simultaneously interplay with neutrophils, promoting NETs formation. NETs provide a scaffold for platelets and induce activation and aggregation of platelet via their complex components, thus generating a positive feedback cycle to each other.

### NETs as a valuable marker in cancer from the clinical perspective

Several techniques for the detection of NETs showed promising clinical applications for diagnosis, therapeutic response, and prognosis. ELISA technique was the most commonly acknowledged to detect the circulating NETs-associated complexes, allowing the quantitative assessment of NETs. In certain studies, the circulating level of NET-derived DNA was measured as MPO-DNA, NE-DNA, or circulating DNA [11, 106–108]. Apart from NET-related DNA complex, the circulating H3Cit level was also identified as the number of NETs [109, 110]. Meanwhile, circulating MPO-DNA, NE-DNA, and H3Cit were more specific for NETs quantification than circulating DNA alone [107]. Based on the evidence of a cohort of 283 gastric adenocarcinoma (GAC) patients, it seemed that both the serum and plasma of blood samples could all be employed for NETs detection [108]. The immunohistochemical (IHC) technique was also used to measure NET formation in the primary tumor lesion or metastatic site of the tumor tissue sample [79, 111]. The NET formation was identified as the neutrophils positive for the H3Cit signal [112, 113]. In some cases, the NETs level, measured as other NETs-specific proteins like MPO, NE, and so on, has been applied as a surrogated marker of NETs [10, 13, 103].

The increment of circulating DNA in plasma, considered a specific marker of NETs in this research, was founded in cancer-related stroke patients [114]. Nevertheless, this data should be cautiously interpreted due to the circulating DNA also involving in the apoptotic, necrotic, and so on [115]. NET-derived proteins like MPO or NE could bind to DNA in the circulating system. For both esophagogastric and lung adenocarcinoma, the level of circulating MPO-DNA was elevated compared to healthy people [106]. According to the analysis of pancreatic adenocarcinoma patients, the level of circulating MPO-DNA before treatment was correlated positively with the clinical stage [19]. The expression of serum MPO-DNA was validated as a predictor of liver metastasis for early-stage breast cancer patients [11]. Tohme S et al*.* revealed that metastatic colorectal cancer patients with elevated levels of serum MPO-DNA after liver resection surgery were more likely to have a reduction in disease-free survival (DFS) [42]. Yazdani HO et al*.* also investigated that the pre-operatively serum MPO-DNA complexes levels increased in proportion to the clinical outcome, observing the added NETs level in patients with shorter DFS and overall survival [41]. In addition, the serum MPO-DNA complex level was confirmed to monitor the HER2 inhibitor-associated vasculitis activity from a prospective cohort of breast cancer [116]. Compared with localized breast cancer, the levels of plasma NE-DNA complexes were higher in regional and distant stages [117]. As a specific NETs biomarker, serum NE-DNA showed better diagnostic efficiency compared with other common clinical biomarkers in gastric Adenocarcinoma, like carbohydrate antigen 19–9 (CA19-9) and carcinoembryonic antigen (CEA). It was identified that serum NE-DNA increased along with the existence of lymph node metastasis. The baseline serum level of NETs was inversely correlated with PFS for GC patients with negative HER2 status. Prompted by the above evidence, NETs quantified by NE-DNA complexes could be identified as an effective diagnostic and prognostic risk factor value in GC [108].

Citrullinated histone H3 (H3Cit) is a representative marker of chromatin decondensation during the NET formation process. It was reported that a significant increment in circulating H3Cit was directly associated with poor clinical outcomes in a panel of tumors [109]. Grilz E et al*.* collected the venous blood samples of 957 patients with cancer and performed a median of 666 days follow-up, revealing that the increased plasma H3Cit level was in an independent correlation with higher cancer mortality (HR = 1.1, *P* < 0.001) [110]. In a cohort enrolling 317 pancreatic ductal adenocarcinoma patients, the amounts of tumor-infiltrating NETs which were quantified by the IHC staining for H3Cit was correlated with RFS and OS, regardless of the state of neutrophil infiltration in the tumor. What’s more, combined with NETs, the diagnostic accuracy was improved by the TNM staging system in pancreatic cancer [113].

In our previous analysis, we first constructed a panel of genes signature as NET-score using the RNA-sequencing data of The Cancer Genome Atlas (TCGA) pan-cancer cohort and measured the NETs density in tumor tissue through IHC validation. NET was correlated with unfavorable prognosis in most types of cancer, like Kidney renal clear cell carcinoma (KIRC), Lung adenocarcinoma (LUAD), and Colon adenocarcinoma (COAD) [13]. In summary, NETs exert a fundamental role as a marker from the clinical perspective (shown in Table 2). The above studies provide the value of NETs as a biomarker in cancer with several NET-associated metrics. From a technical point of view, the main difficulty in the clinical application of NETs was the lack of unified detection metrics and standard quantification threshold.

**Table 2:** NETs as a valuable marker in Cancer from the clinical perspective

Year	Method	Samples number	Chosen markers of NETs	Type of Cancer	Function	Reference
2016	ELISA	35	MPO-DNA complex	Colorectal cancer	correlated with the reduction in DFS	[42]
2018	ELISA	161	Citrullinated histone H3	Pan-cancer	prognostic blood marker	[109]
2019	ELISA	45	NE-DNA complex	Breast cancer	associated with clinical stages	[117]
2019	ELISA	27	MPO-DNA complex	Colorectal cancer	correlated with shorter survival	[41]
2019	ELISA	104	MPO-DNA complex	Pancreatic ductal adenocarcinoma	related to the clinical stage	[19]
2019	ELISA	957	Citrullinated histone H3	Pan-cancer	associated with higher mortality	[110]
2019	IHC	317	Citrullinated histone H3	Pancreatic ductal adenocarcinoma	prognostic factor	[113]
2019	ELISA	138	circulating DNA	Pan-cancer	related to cancer-related stroke	[114]
2019	ELISA	75	MPO-DNA complex	Esophagogastric and lung adenocarcinoma	correlated with advanced stage	[106]
2020	ELISA	356	NE-DNA complex	Gastric Adenocarcinoma	diagnostic, therapeutic predictive, and prognostic value	[108]
2020	ELISA	461	MPO-DNA complex	Breast cancer	metastases predictor	[11]
2022	IHC	321	MPO	Pan-cancer	prognostic factor	[13]
2022	ELISA	25	MPO-DNA complex	Breast cancer	monitoring HER2 antibody–drug-associated vasculitis activity	[116]

### The synergistic effect of NETs inhibition combined with immunotherapy

There is a growing study focusing on the mechanism of the immunosuppressive environment in cancer, especially after the clinical application of immune checkpoint inhibitors (ICIs) with promising therapeutic effects. The goal of ICIs is to reactivate the immunity response and rescue the anti-tumor effects in cancer. Although a certain percentage of patients showed a favorable benefit from the ICIs treatment, how to improve the response rate and relieve the adverse effects of treatment is always a challenge for clinicians. It was founded that the inhibition of NETs maybe has a synergistic effect with ICIs for cancer treatment [118]. Zhang Y et al*.* further investigated that increased CD8^+^ T cells were recruited in the mouse deficient of PAD4. And the pancreatic implantation tumor showed a significant reduction with the additional employment of PD-1 blockade in the mouse deficient in PAD4 (PAD4-KO) [119]. The PAD4 inhibition of NETosis has also been described with the potential synergistic effect with ICIs through the modulatory role for lymphocyte function, like T and NK cells [28]. The implantation of melanoma cells in mice resulted in tumor regression with anti-PD1 or anti-CTLA4 immunotherapy, and the increment of NETs during treatment was speculated in association with adverse reactions [118]. In addition, the current research on the impact of NETs on immunotherapy is mainly carried out in vitro or in vivo. There is a lack of relevant evidence for the role of NETs on immunotherapy in humans. We also intend to explore the relationship between NET and immunotherapy efficacy and adverse effects in the patient cohort of tumor immunotherapy. Whether the use of NETs inhibitors can improve the response rate of immunotherapy and relief adverse reactions deserves further exploration. Therapeutic strategies targeting NETs combined with immunotherapy treatment may be a promising regimen with improved treatment benefits in the future.

### Potential treatment strategies for inhibiting NETs

Since plenty of shreds of evidence have indicated that NETs might play vital roles in different diseases, including inflammation injury, sepsis, auto-immune disease, and cancer, targeting NETs is now expected to be a potential treatment strategy. Several works which are under experimental research or preclinically used include degrading already formed NETs and blocking the aberrant formation of NETs (shown in Table 3).

**Table 3:** Potential targets for NETs inhibition in different disease

Agent/Inhibitor	Target/Function	Disease/Pathology	Subject of research	Reference
DNase I	Degrading formed NETs	Diabetic wound healing	Diabetic mouse	[120]
		Liver IRI	Liver IRI mouse	[121]
		lung injury/ARDS	MRSA-infected mouse	[122]
		SLE	Serum of SLE patients	[123, 124]
		Scar formation	Mouse model of laminectomy	[67]
		Cystic fibrosis	Cystic fibrosis patients	[125, 126]
		Colorectal cancer	MC38-bearing mouse	[127]
		Hepatocellular carcinoma	Hepa1-6 or HuH7 bearing mouse	[43, 128]
		Breast cancer	SCP28-bearing mouse	[129]
		Pancreatic cancer	Panc02-bearing mouse	[19]
rhDNase I	Degrading formed NETs	COVID-19 Infection	Patients in Phase I clinical trial	NCT04409925
GSK484	Inhibitor of PAD4	Renal IRI	Renal IRI mouse	[130]
		Lung injury	SAH mouse	[131]
		Hepatocellular carcinoma	Hepa1-6 bearing mouse	[132]
		Breast cancer	4T1-bearing mice	[28, 129]
Cl-amidine	Inhibitor of PAD4	Mastitis	LPS-induced mouse mastitis	[133]
		Endometritis	LPS-induced rat endometritis	[134]
BMS-P5	Inhibitor of PAD4	Multiple myeloma	DP42-bearing mouse	[135]
Sivelestat	Inhibitor of NE	Ovarian cancer	Human neutrophils	[136]
		Breast cancer	SCP28-bearing mouse	[129]
GW311616	Inhibitor of NE	Diffuse large B-cell lymphoma	A20-bearing mouse	[21]
		Inflammatory responses	Human neutrophils	[137]
AZD5904	Inhibitor of MPO	Multiple organ dysfunction in sepsis	Human neutrophils	[138]
Reparixin	CXCR1/2 inhibitor	solid malignancies	Tumor-bearing mice	[28]
Danirixin	CXCR2 antagonist	COPD	Patients in Phase II clinical trial	NCT03250689
Metformin	Inhibiting NADPH oxidase activity	SLE	Proof-of-Concept Trial	[139]

DNase I is one of the endonucleases that cleave DNA, resulting in the collapse of the web-like structure, and is commonly used as a NETs inhibitor [140]. Previous studies reported that Dnase I could improve diabetic wound healing through the clearance of NETs [141] and mechanistically research revealed that Dnase I exerted its function mainly by improving inflammation resolution, reactivating epithelial regeneration-related signaling pathways, and attenuating the cumulation of reactive oxygen species (ROS) [142]. Timely elimination of excessive NETs is crucial for tissue homeostasis and avoiding the presentation of self-antigens [123]. Evidence showed that inhibiting NET formation by the treatment of Dnase I significantly protected hepatocytes and reduced inflammation after liver ischemia/reperfusion (I/R) injury [121], lessened lung injury, and improved survival both in mouse models and in humans with ARDS from pneumonia or sepsis [122]. Impairing the function of Dnase I failed to remove NETs in time might result in the pathogenesis of lupus nephritis in systemic lupus erythematosus (SLE) patients [124]. Treatment with DNase I might be a therapeutic target in SLE, which still demanded further research [123, 124]. In a mouse spine operation model, the authors proved that NETs promoted scar formation in post-epidural fibrosis, which was remarkably decreased with the administration of DNase I [67]. Moreover, recombinant Dnase I is used for the treatment of cystic fibrosis currently due to its inhibitory function in NETs [125, 126]. Emerging evidence revealed that NET formation favors tumor cell proliferation, metastasis, as well as immunosuppression [115, 143]. Degrading NETs by Dnase I not only reduced circulating NET levels, but also suppressed tumor cell growth and metastasis in colorectal cancer, hepatocellular carcinoma, breast cancer, and pancreatic cancer [19, 127]. PAD4 catalyzes the citrullination of histones and promotes chromatin decondensation during the formation of NETs [144, 145]. Inhibition of PAD4 is an effective method for NETs suppression and is under research in different diseases [144]. Inhibiting PAD4 by GSK484 reduced NETs formation, and was proved to improve renal function via apoptosis limitation in a mouse IRI model [130], and could attenuate the swelling of the alveolar interstitium caused by a subarachnoid hemorrhage in mouse [131]. Moreover, GSK484 decreased tumor lung metastasis in hepatocellular carcinoma and breast cancer mouse models due to NETs inhibition [28, 129, 132]. Besides GSK484, Cl-amidine and BMS-P5 are also PAD4 inhibitors that are under investigation [135, 146]. In an LPS-induced mouse mastitis model, Cl-amidine reduced NETs release and pathological injury, which might be relevant to inhibiting NF-κB, MAPK, and NLRP3 signaling pathways [133]. It was reported that Cl-amidine could also weaken the inflammatory response of LPS-induced endometritis in rats by decreasing the formation of NETs [134]. BMS-P5 is a novel PAD4-specific inhibitor. A recent study reported that BMS-P5 blocked citrullination of histone H3 and NETs formation in human multiple myeloma (MM) cells, and treatment with BMS-P5 to MM-bearing mice attenuated symptoms and disease progression [135].

The protease NE and MPO are vital components of NETs, and they work synergistically to decondense chromatin in NETs [147]. Some studies reported that NE or MPO deficiency fails to produce NETs both in vitro and in vivo [148, 149]. Inhibition of these two enzymes would likely serve as therapeutic targets in NET-associated diseases [149]. Sivelestat is a NE inhibitor that has been approved to treat ARDS in Japan and South Korea [115]. The generation of NETs proves to be beneficial for pro-migratory tumor behavior. In a three-dimensional (3D) model that mimics a tumor-immune microenvironment, the authors demonstrated that inhibiting NETs formation by sivelestat prevents ovarian tumor cells from acquiring an invasive phenotype [136]. A recent study reported that CXCR2 favored NETs formation by enhancing the recruitment of TANs towards brain metastasis in breast cancer cells, and it could be impeded through sivelestat administration [150]. Reparixin as a CXCR1/2 inhibitor could impede NETosis [28]. GW311616 is another NE inhibitor that could attenuate the proliferation and migration of diffuse large B-cell lymphoma cells induced by NETs [21]. Minerals-organic particles in the human body were shown to promote pro-inflammatory responses via NETs which could also be blocked by GW311616 [137]. Strategies to impede NETs by the inhibitors of MPO are also effective. Excessive release of NETs in septic patients and high levels of NETs-MPO in septic patients were relevant to the severity of organ dysfunction. AZD5904, an inhibitor of MPO could reduce the formation of NETs and therefore may be a new therapeutic option for multiple organ dysfunction in sepsis [138].

Fortunately, phase I (NCT04409925) and phase II (NCT03250689) clinical trials inhibiting NETs are being carried out in non-neoplastic diseases. Once successful, it will lay the foundation for clinical trials in cancer.

## Conclusion and the future perspective

**Fig. 1:**
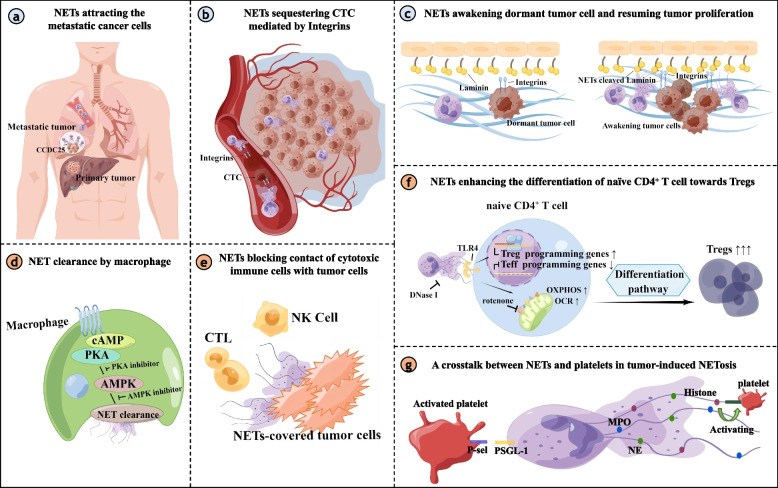
Intercellular communication between NETs and tumor cells or immune cells. **a** NET-DNA served as a chemotactic factor, which was sensor by cell-surface receptor CCDC25 on the metastatic tumor cell, and subsequently activated the downstream ILK–β-Parvin signaling to accelerate the metastasis.** b** NETs functioning as a substrate for the integrins expressed on the tumor cell, could enhance the cell adhesion ability and sequester the CTC.** c** NET-remodeled laminin-111 contributed to tumor proliferation by activating the integrin a3β1 signaling of the dormant cancer cell. **d** NET clearance refers to the process by which macrophage phagocytoses the NET. NET clearance is often impaired in cases of abnormal immunity. Specific molecules could enhance the ability of NET clearance through the cAMP/PKA/AMPK axis. **e** The impact of NETs on the immunomodulatory function may be due in part to the phenomenon of NETs-shielding tumor cells, reducing the direct contact between effector cytotoxic lymphocytes and tumor cells. **f** NET-primed naïve CD4^+^ T cell was more inclined to the differential of Treg cell through increasing the mitochondrial OCR and OXPHOS. Genes essential for Treg differentiation and activity were up-regulated. Meanwhile, Teff programming genes were significantly down-regulated. **g** Activated platelets bind to the receptor P-selective (P-sel) glycoprotein ligand-1 (PSGL-1) on the surface of the neutrophil via P-selective protein and promote the production of NETs. And NETs such as the DNA backbone and adherent thrombosis-related enzymes can activate platelets in turn

Given that neutrophils are core innate immune signaling hubs that could transmit or activate most of the pathways indispensable for adaptive immune activities, there is a growing interest in the research of neutrophils, especially for NETs. The prior reviews mainly discussed the crucial role of NETs in contributing to the development and progression of cancer [115, 143, 151–153]. Our review illustrated the altered immune networks mediated by NETs in cancer. Based on the available data, we focused on the intercellular communication between NETs, cancer cells, and immune cells (Fig. 1). This information is the key issue to figuring out the specific molecular mechanism of NET-cell contact for targeted therapy and therapeutic intervention. Meanwhile, it remains much left to delineate the NETs-primed molecular networks in cancer. Previously, the focus in the field of tumor immunology was mostly on lymphocytes, but now there is increasing evidence that the role of neutrophils is indispensable. For example, the neutrophil-to-lymphocyte ratio (NLR) can serve as an independent prognostic factor and predict the efficacy of immunotherapy. Therefore, it is necessary to further understand neutrophils. One of the key limitations is that primary human neutrophils have a short survival time with only several days in vitro. Neutrophils could not be transfected to inhibit the specific signaling pathway in association with NETs formation and function. Most advanced technologies, like sc-RNA sequencing or labeling system, have been highly adaptable to ligand/receptor interactions. Nevertheless, whether these technologies could be a technology in the application for deeply resolving cellular interaction of NETs deserves further validation. We highlighted the recent research in our review that the inhibition of NETs exerted a synergistic part combined with ICIs immunotherapy in preclinical studies. Due to their connection between innate and adaptive immune response, we aspired to supply a novel framework regarding NETs targeted inhibitors combined with other immunotherapy strategies to improve clinical treatment benefits for cancer.

## Data Availability

Not applicable.

## Abbreviations

NETs: Neutrophil extracellular traps
TANs: Tumor-associated neutrophils
MPO: Myeloperoxidase
TLRs: Toll-like receptors
PKC: Protein kinase C
NE: Neutrophil elastase
PAD4: Peptidyl arginine deiminase 4
TME: Tumor microenvironment
EMT: Endothelial-to-mesenchymal transition
CTCs: Circulating tumor cells
scRNA-seq: Single-cell RNA sequencing
PAMP: Pathogen-associated molecular pattern
PGC1-α: Peroxisomes proliferator-activated receptor gamma coactivator 1-alpha
DLBCL: Diffuse large B-cell lymphoma
IL-8: Interleukin-8
CXCR2: C-X-C Motif Chemokine Receptor 2
HCC: Hepatocellular carcinoma
COX2: Cyclooxygenase-2
ILK: Integrin-linked kinase
MMP9: Matrix metalloproteinase 9
FAK: Focal adhesion Kinase
ERK: Extracellular signal regulated kinase
YAP: Yes-associated Protein
IL-1β: Interleukin-1β
IL-6: Interleukin-6
TNF: Tumor necrosis factor
TAMs: Tumor associated macrophages
ETs: Extracellular traps
MET: Macrophage extracellular trap
pNET: Pancreatic neuroendocrine tumor
RFS: Recurrence-free survival
ARDS: Acute respiratory distress syndrome
LDG: Low density granulocyte
COVID-19: Coronavirus disease 2019
EDIL3: EGF-like repeats and discoidin I-like domain 3
LFA1: Lymphocyte function-associated antigen 1
ICAM: Intercellular adhesion molecule
Mertk^−^MHC-II^lo^^−^^int^: Myeloid-epithelial-reproductive tyrosine kinase/major histocompatibility complex II
MI: Myocardial infarction
RvTs: 13-Series resolvins
cAMP: Cyclic adenosine monophosphate
PKA: Protein kinase A
AMPK: AMP-activated protein kinase
NK: Natural killer
NSCLC: Non-small cell lung cancer
BC: Bladder cancer
Tregs: Regulatory T cells
OXPHOS: Oxidative phosphorylation
Teff: Effector T cell
GC: Gastric adenocarcinoma
IHC: Immunohistochemical
DFS: Disease-free survival
CA19-9: Carbohydrate antigen 19–9
CEA: Carcinoembryonic antigen
H3Cit: Citrullinated histone H3
TCGA: The Cancer Genome Atlas
KIRC: Kidney renal clear cell carcinoma
LUAD: Lung adenocarcinoma
COAD: Colon adenocarcinoma
ICIs: Immune checkpoint inhibitors
ROS: Reactive oxygen species
I/R: Ischemia/reperfusion
SLE: Systemic lupus erythematosus
MM: Multiple myeloma
NLR: Neutrophil-to-lymphocyte ratio
